# Space–Time–Frequency Multi-Sensor Analysis for Motor Cortex Localization Using Magnetoencephalography

**DOI:** 10.3390/s20092706

**Published:** 2020-05-09

**Authors:** Vincent Auboiroux, Christelle Larzabal, Lilia Langar, Victor Rohu, Ales Mishchenko, Nana Arizumi, Etienne Labyt, Alim-Louis Benabid, Tetiana Aksenova

**Affiliations:** 1 Univ. Grenoble Alpes, CEA, LETI, CLINATEC, MINATEC Campus, F-38000 Grenoble, France; vincent.auboiroux@cea.fr (V.A.); christelle.larzabal@cea.fr (C.L.); victor.rohu@gmail.com (V.R.); ales.mishchenko@mail.ru (A.M.); arizumi@gmail.com (N.A.); etienne.labyt@cea.fr (E.L.); alimlouis@sfr.fr (A.-L.B.); 2 CHU Grenoble Alpes, CLINATEC, F-38000 Grenoble, France; llangar@chu-grenoble.fr

**Keywords:** magnetoencephalography, cortex, source imaging, localization, time–frequency, multi-sensor, linear regression, coefficient of determination

## Abstract

Brain source imaging and time frequency mapping (TFM) are commonly used in magneto/electro encephalography (M/EEG) imaging. However, these methods suffer from important limitations. Source imaging is based on an ill-posed inverse problem leading to instability of source localization solutions, has a limited capacity to localize high frequency oscillations and loses its robustness for induced responses (ill-defined trigger). The drawback of TFM is that it involves independent analysis of signals from a number of frequency bands, and from co-localized sensors. In the present article, a regression-based multi-sensor space–time–frequency analysis (MSA) approach, which integrates co-localized sensors and/or multi-frequency information, is proposed. To estimate task-specific brain activations, MSA uses cross-validated, shifted, multiple Pearson correlation, calculated from the time–frequency transformed brain signal and the binary signal of stimuli. The results are projected from the sensor space onto the cortical surface. To assess MSA performance, the proposed method was compared to the weighted minimum norm estimate (wMNE) source imaging method, in terms of spatial selectivity and robustness against an ill-defined trigger. Magnetoencephalography (MEG) recordings were performed in fourteen subjects during two motor tasks: finger tapping and elbow flexion/extension. In particular, our results show that the MSA approach provides good localization performance when compared to wMNE and statistically significant improvement of robustness against ill-defined trigger.

## 1. Introduction

Magnetoencephalography (MEG) is routinely used in non-invasive dynamic functional brain imaging. Functional brain imaging is commonly performed using source imaging methods. The basic principle of source imaging involves the use of the dipolar biophysical model to reconstruct brain sources from scalp measurements, assuming that the MEG signal can be expressed as a linear mixture of brain sources in the presence of noise [1]. The time course of each dipole current is estimated from the MEG signal. Currently, brain source imaging uses a high number of dipoles (typically around 15,000) in order to reconstruct the brain activity. Consequently, the number of dipoles generally exceeds the number of sensors (up to 306). This results in an ill-posed inverse problem that requires a regularization step [2]. Several methods have been developed to deal with this, including minimum norm, Bayesian, tensor, sparse, and subspace based approaches [3,4,5,6]. Priors used in regularizing the inverse problem apply to spatial, temporal, or spatio-temporal distribution of the sources, resulting in a tradeoff between temporal or spatial source imaging resolution.

Classically, sources are computed by averaging brain signals from many trials to increase the signal to noise ratio [7]. However, trial averaging is generally sensitive to ill-defined event triggers, which occur for induced response studies, due to variable subject response time. Motor triggers are often used in MEG studies to synchronize the brain activity to the actual motor execution. However, in some cases, such as in Brain Computer Interface (BCI) studies involving mental tasks and/or patients with motor disabilities, motor triggers cannot be measured [8]. In this case, visual cues triggered on the timing of the motor instruction could be used with a compromise on timing accuracy.

Another limitation of approaches to source imaging concerns high frequency brain oscillation localization, i.e., the averaging results in low pass filtering of brain signals. Subsequently, identified brain sources mainly represent low frequency brain oscillations. Sources of high frequency oscillation have been reported in epilepsy or for evoked activity [9,10,11,12]. However, these cases correspond to synchronous oscillations. Moreover, these high frequency sources are localized from a limited number of frequencies, usually one or a few narrow frequency bands pre-selected from a time frequency (TF) analysis in sensor space. TF analysis has also been performed by using cortical source reconstructed MEG data [13,14,15]. However, these TF analyses integrated to cortical source reconstructed MEG data also provide limited frequency resolution.

Regarding the brain signal, to take into account its transient and non-stationary characteristics, the most relevant processing approach is TF analysis. The TF content of a brain signal is particularly important for a number of applications, e.g., epilepsy seizure detection/prediction, movement disorders, brain computer interface (BCI) studies. TF brain signal content is used for time–frequency mapping (TFM), which is primarily applied in MEG data analysis [16,17,18]). TFM is the simplest way for TF analysis of MEG data in sensor space. TFM averages TF transformed signals across trials, according to stimuli. The results are scaled to the mean energy of the signal at each frequency. The modulation of brain activity is expressed as a percentage of amplitude change according to the baseline activity for a given sensor, frequency, and time delay related to stimuli [19]. TFM is used to identify informative frequencies and to localize task-related modulations of brain activity [20]. The drawback of TFM is that it involves the independent analysis of signals from a number of frequency bands, and from co-localized sensors (namely, the use of an Elekta MEG system with a triplet of co-localized sensors). This results in several criteria associated with the same location. Nevertheless, estimation of the general level of signal modulation, for a given location, across a set of frequencies, is desirable for a number of applications.

In the present article, we propose a new methodological approach to overcome the following drawbacks of widely applied source imaging and TFM: (a) sensitivity to ill-defined triggers; (b) limited frequency resolution, and particularly, limited capability to localize high frequency oscillations; (c) potential instability caused by ill-posed inverse problem of source imaging, and (d) difficulties in processing multi-sensor/multi-frequency data by TFM. Although the proposed method is especially suited to the analysis of MEG signals acquired with co-localized sensors (Elekta MEG device, Elekta, Helsinki, Finland), this method can be applied more largely to integrate multi-frequency information, or data from different kinds of sensors (e.g., EEG and MEG).

The proposed multi-sensor space–time–frequency analysis (MSA) consists of a simple regression-based statistical method. TF transformed MEG signals from co-localized sensors are considered as inputs, and shifted binary stimuli signal is considered as output. Shifted multiple Pearson correlation (MPC) is calculated for input and output signals, using linear regression. This allows the co-localized sensors and/or multi-frequency information to be integrated. The MPC of shifted binary stimuli, and prediction from regression, characterizes the general signal modulation in a sensor space, for a given location and for a given delay (shift) related to stimuli. To prevent overtraining, which may result in overestimation of MPC, a cross-validation procedure is integrated in to the MPC estimation procedure. In addition, L2 regularization is applied to stabilize the MPC in the presence of correlated variables. Further, MSA cortical maps are obtained by projecting results from the sensor space onto the brain surface.

The proposed MSA method has been compared to the weighted minimum norm estimate (wMNE) source imaging method, in terms of spatial selectivity and robustness against an ill-defined trigger, namely a visual trigger instead of a motor trigger. wMNE was chosen, as this method is widely used in MEG source imaging and is classified as the gold standard. In our study, brain activity was recorded with MEG during upper limb tasks (finger tapping, elbow flexion) in healthy subjects. The study involved fourteen subjects, and data were analyzed using both approaches.

## 2. Materials and Methods

### 2.1. Ethics Statement

This study was carried out in accordance with the recommendations of good practice for conducting and reporting MEG research [21]. The study was approved by the local Ethical Committee (CPP) and the French National Agency for Medicines and Health Products Safety (ANSM) (Clinical trial n°: NCT02574026, CPP n°: 10-CHUG-7, ANSM id RCB n°: 2010-A00421-38 and clinical trial n°: NCT02790411, CPP n°: 13-CHUG-07, ANSM id RCB n°: 2013-A00414-41). All the subjects gave written informed consent in accordance with the Declaration of Helsinki before experimentation.

### 2.2. Participants and Tasks

Overall, 14 subjects with unknown neurological and psychiatric disorders were included in the study. Two experiments involving motor movements are considered. In the first experiment referred to as “FingerTap”, seven participants (female = 5/7, mean age = 37 y, SD = 17 y) were asked to press a pad using their right index finger. In the second experiment, called “FlexElbow” seven participants (female = 3/7, mean age = 27 y, SD = 8 y) were asked to perform flexions of the right elbow. For these two experiments the participants were sitting down in front of a stimulus screen while their MEG activity was recorded.

The “FingerTap” experiment offers two different triggers to analyze the brain activations associated to motor activations: 1/a motor trigger which provides a precise timing of the movement onset, 2/a visual cue which was used to display the movements instructions to the participants. This is different from the “FlexElbow” experiment for which trials could only be analyzed based on the visual cue as no motor trigger was available. 

**Figure 1:**
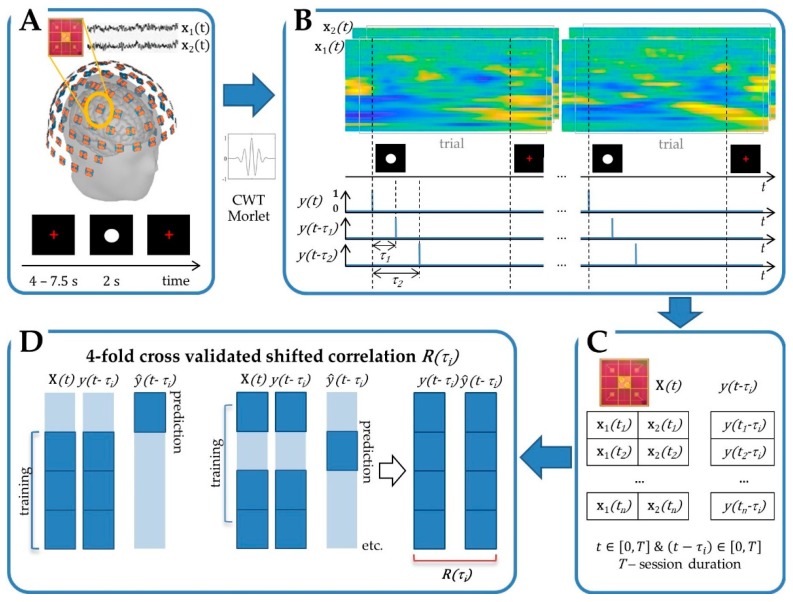
Cross-validated shifted multiple correlation is calculated in sensor space. (**A**) Neural signal recording with Elekta Neuromag system comprising 306 measurement channels organized in channel-triplets (two planar gradiometers and one radial magnetometer, not used in this study), on 102 silicon chips, in a helmet-shaped array. (**B**) Spectral decomposition using Morlet continuous wavelet transform (CWT) and feature extraction. (**C**) Matrices of features (e.g., for a channel-doublet of two gradiometers) and shift ($\tau_{i}$) for a given session, including trials and basic activity periods. (**D**) Four-fold cross-validated shifted multiple correlation.

Each trial started with the presentation of a red fixation cross of varying duration (2.25–4 s) followed by a visual cue (white sphere) which was displayed for two seconds (Figure 1A). Prior to the session, the participants were instructed about the movement to be executed every time the visual cue appeared on the screen. For each experiment, the stimuli (n > 100 trials) were presented using Stim2 software (Compumedics, Victoria, Australia). The duration of a session was approximately 10 min.

Throughout the course of recording, the subjects were video-monitored and could communicate with the experimenter via a MEG-compatible intercom system.

### 2.3. MEG Measurements

Participants’ MEG activity was measured in a magnetically shielded room using a 306-channel whole scalp array (204 planar gradiometers and 102 magnetometers) from the Elekta Neuromag system (Elekta Neuromag Oy, Helsinki, Finland). ECG and EOG were recorded simultaneously. The recording sampling rate was 1000 Hz. Continuous head position indicator (cHPI) signals were recorded during the experiments to track the subject’s head movements.

Prior to experimentation, a 3D digitization system (Isotrak II^®^, Polhemus, Colchester, VT, USA) was used to localize anatomical fiducial points for later co-registration with magnetic resonance acquisition (MRI). The relative positions of the cHPI emitters and of numerous points over the participant’s head surface were also measured. For the “FingerTap” experiment, a fiber optic-based sensor allowed the recording of the participant’s index tapping. It provided the onset/offset timing of the executed movement.

Individual anatomical 3D T1-weighted magnetic resonance acquisitions were performed using a 1.5 T MRI scanner for the “FingerTap” subjects (Magnetom Espree, Siemens, Erlangen, Germany) and a 3 T MRI scanner for the “FlexElbow” subjects (Achieva TX, Philips, Eindhoven, The Nederlands), with a resolution of 1 × 1 × 1.1 mm.

### 2.4. Pre-Processing

Temporal signal space separation (tSSS) [22] was applied to reduce the noise in the MEG data using MaxFilter 3.0 software (Elekta, Helsinki, Finland). First, a manual reviewing of raw data allowed the marking of bad channels. Second, the tSSS filter was applied using head movement compensation and automatic bad channel correction. The main parameters were kept to default (tSSS correlation threshold of 0.98, orders of expansion for “in” and “out” components of signal respectively set to 8 and 3, and a 10 s-long time buffer). Notch filtering at 50 Hz and harmonics (100 Hz, 150 Hz, 200 Hz and 250 Hz) was also applied to remove power line contamination.

### 2.5. Anatomy

Grey and white matters were segmented, scalp and cortical surfaces were reconstructed and the parcellation of their folding pattern was calculated using the “recon-all” pipeline available in the FreeSurfer software package (http://surfer.nmr.mgh.harvard.edu, [23]). MRI data, meshes for scalp and cortex surfaces and cortical surface parcellation were imported in Brainstorm software (http://neuroimage.usc.edu/brainstorm/, [19]). Fiducials and digitized head points were used to co-register the anatomical MRI scans with the MEG recording. 

**Figure 2:**
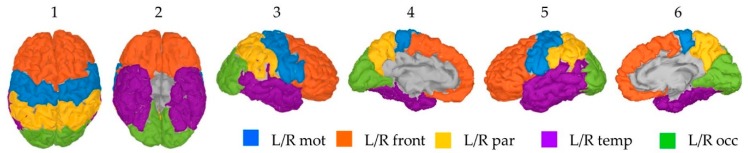
Anatomical location of the selected ten region for a given participant—1: frontal, 2: bottom, 3: right external, 4: left internal, 5: left external and 6: right internal view.

For the two hemispheres, participants’ cortical surface was divided according to the four external lobes: frontal, parietal, temporal and occipital. Because in this study we were particularly interested in activations linked to motor movements, the motor area located in the posterior part of the frontal lobe was isolated as a separate region. Ten distinct areas namely the left/right motor region (Lmot/Rmot), the left/right frontal lobe anterior to the motor region (Lfront/Rfront), the left/right parietal lobe (Lpar/Rpar), the left/right temporal lobe (Ltemp/Rtemp) and the left/right occipital lobes (Locc/Rocc) are therefore considered in this study. These regions were created by merging the specific cortical parcellations obtained from the FreeSurfer’s Destrieux surface-based atlas (Figure 2). 

### 2.6. MSA

#### 2.6.1. Spectral Decomposition

For spectral decomposition, a complex continuous wavelet transform (Morlet) was applied for each sensor, in 30 frequency bands spread regularly in a logarithmic scale ranging from 1 Hz to 250 Hz. Morlet wavelet was chosen as it is a popular tool for time–frequency decompositions of electrophysiological data [19,24], and is particularly used in BCI applications [25,26]. The absolute values of wavelet coefficients were averaged in sliding windows (300 ms), centered, and then scaled to the energy of the signal at each frequency [27].

#### 2.6.2. Multiple Coefficient of Correlation

Conventional TFM averages the task-related TF decomposed signal of brain activity from each sensor across the trials, and refers it to the basic brain activity. Similarly, the shifted Pearson correlation (PC) $R_{f}\left(\tau \right)$ can be calculated.

Let us consider

- $x_{f}\left(t\right)$, a spectral component of the brain signal, f is a frequency;
- y(t), a binary marker of stimuli: y(t)=1 at the stimulus time occurrence and y(t)=0 otherwise; stimuli correspond to the starting points of trials (Figure 1).

Here time t ∈ [0,T] and T is session duration.

Shifted PC of y(t) and the centered $x_{f}\left(t\right)$ for a time shift $\tau \;\in \;\left[0,\tau_{max}\right]$ is estimated as [28]:

$$R_{f}\left(\tau \right)=\frac{1}{n}\sum_{t}x_{f}\left(t\right)\left(y\left(t-\tau \right)-\bar{y}\right)/\sigma_{x_{f}}\sigma_{y}.$$

Similar to TFM, $R_{f}\left(\tau \right)$ characterizes the difference between averaged event-related brain activities and basic brain activities, for a spectral component. Simple PC is calculated for bivariate data: scalar $x_{f}\left(t\right)$ and y(t). It characterizes task-related signal modulation at moment $\tau \in \left[0,\tau_{max}\right]$ related to the beginning of a trial, for one sensor and one frequency. On the contrary, multiple PC (MPC) R(τ) can be estimated for multivariable data. MPC is the square root of the coefficient of determination [28], which estimates the fraction of the variance in the output variable y(t) that is explained by the vector of inputs x(t), using linear regression

$$\hat{y}\left(t\right)=b^{T}x\left(t\right).$$

Here, coefficient b is the least square estimate. MPC can be calculated for the brain activity recorded by the co-localized sensors (e.g., pair of gradiometers $x_{1}\left(t\right)$, $x_{2}\left(t\right)$) and/or for a set of frequencies $\left\{f_{1},f_{2},\ldots \right\}$. The input vector of brain activity x(t) summarizes the signals recorded during a session at a given location (all sensors), and is analyzed in a set of frequencies:

$$x\left(t\right)={\left(x_{1}^{T}\left(t\right),x_{2}^{T}\left(t\right)\right)}^{T};\;x_{i}\left(t\right)={\left(x_{i,f_{1}}\left(t\right),x_{i,f_{2}}\left(t\right),\ldots \;\right)}^{T};\;i=1,2;\;f_{i}\in \left\{f_{1},f_{2},\ldots \right\};\;\;t\in \left[0,T\right].$$

Similar to simple PC, shifted stimuli y(t−τ) are considered as the output (Figure 1). The shifted MPC R(τ) characterizes the overall modulation of brain activity (all sensors, all frequencies) at a given location in sensor space, at time moment τ, in relation to the stimuli.

The MPC is based on least-squares regression. The increase in the dimension, including recordings from several sensors which are decomposed in a set of frequencies, may cause an overfitting effect and overestimation of the correlation level. The n− fold cross-validation procedure is introduced into the MPC calculation to avoid this overfitting. The whole dataset is split into non-overlapping subsets. While n−1 subsets are used to estimate regression coefficients, predicted values $\hat{y}\left(t\right)$ are calculated for the remaining observations. The cross-validation process is repeated n times. Each subset is used exactly once as validation data. R(τ) is estimated from the resulting prediction. Here, four-fold cross-validation was applied (Figure 1).

Another problem associated with multiple regression is correlated variables. Spectral components of a signal can be highly correlated, particularly for neighbor and narrow frequency bins. To overcome the problem, L2-penalization of least squares (ridge regression) [29] is also applied for regression coefficient estimation.

#### 2.6.3. Projection onto the Cortex

To estimate the location of the modulated activity within the cortex, the cortical surface mesh was computed from MRI, as described above. As MPC involves non-linear statistics of observed signals, standard linear inverse methods cannot be applied directly. Therefore, simple projection was used to transpose MPC statistics from sensor space to the brain surface. For this, each point of the brain surface is placed in correspondence to the three nearest sensor loci of the helmet. Then inverse distance weighting (IDW) [30] is locally applied. The assigned values to each unknown point of cortical surface mesh is calculated with a weighted average of the nearest values available at sensor space. The inverse of the distances (Euclidean) from the point of cortical surface mesh to available points at sensor space (“amount of proximity”) is used when assigning weights.

### 2.7. wMNE Source Reconstruction

wMNE was chosen as this source reconstruction method is widely used and well documented in the literature. Moreover, this method has been implemented in a number of open-source software products, such as Brainstorm [19], MNE [31], and Fieldtrip [32], as well as in some commercial software. 

Before wMNE source reconstruction, independent components analysis (ICA) decomposition was calculated using MNE-Python software (v0.18, [31]) using a number of components equal to the data rank. This decomposition was finally loaded in Brainstorm and the components to be removed were manually chosen based on typical time-course and spatial pattern of cardiac and eye-movement artifacts.

Trials from the two experiments were epoched from 0 to 1.5 s post-visual cue onset. For the “FingerTap” experiment trials were also epoched from −0.5 s to 1 s post-movement onset. Trials with multiple responses from subject or residual artifact after tSSS filtering were discarded, using peak-to-peak detection with a threshold set to 5000 fT. The MEG forward model was computed using the analytical approach with multiple nested spheres implemented in Brainstorm software [33,34]. For this step, individual subjects cortical and head surface meshes were used.

Whitened and depth-weighted minimum norm estimates algorithm from Hamalainen’s MNE software, which was implemented in Brainstorm software, was used for the comparison to the MSA statistical approach. This source reconstruction algorithm corresponds to an L2 norm estimate that yields small, distributed estimates of cerebral currents (compared to, for example, an L1 norm, which favors a few, large-amplitude currents) to explain the observed sensor data. The input noise covariance matrix was computed for each subject on a baseline defined between −0.5 and −0.001 s with respect to visual onset, across all trials. Constrained sources were finally reconstructed over the averaged trials.

### 2.8. Group-Level Analysis

Group-level activation maps were calculated using anatomical standardization between participants: all individual wMNE and MSA maps were mapped to the MNI/ICBM152 brain template [35] using the surface-based registration approach available in Brainstorm when individual MRI data is first processed with FreeSurfer. Grand average across participants was finally calculated for each type of reconstruction (wMNE or MSA), each experiment (“FlewElbow” or “TapFinger”) and each trigger when available (visual cue or motor trigger).

### 2.9. Statistical Analyses

For each participant, the mean absolute value over the vertices of the ten regions ((L/R)front, (L/R)mot, (L/R)par, (L/R)temp, (L/R)occ) was computed at each time point of the whole epoch: from 0 to 1.5 s post-visual cue onset and from −0.5 to 1 s post-movement onset. All these values combined across time and region provided a distribution that was specific to each participant. Because they did not necessarily follow a normal distribution, we used a quantile method to detect outlier values that would stand out from the baseline level. For a given region, time point values exceeding the fifth quintile of this distribution (corresponding to a 95% confidence interval in the particular case of a Gaussian distribution) were therefore considered as significant. For each participant, the percentage of significant time points found in the left motor (Lmot) region using the MSA and the wMNE methods was compared by performing one-tailed Wilcoxon signed-rank tests. The same statistical methodology was applied on the group-level data.

## 3. Results

The proposed MSA method was tested on two datasets: the “FingerTap” and the “FlexElbow” experiments in comparison to classical wMNE analyses. 

**Figure 3:**
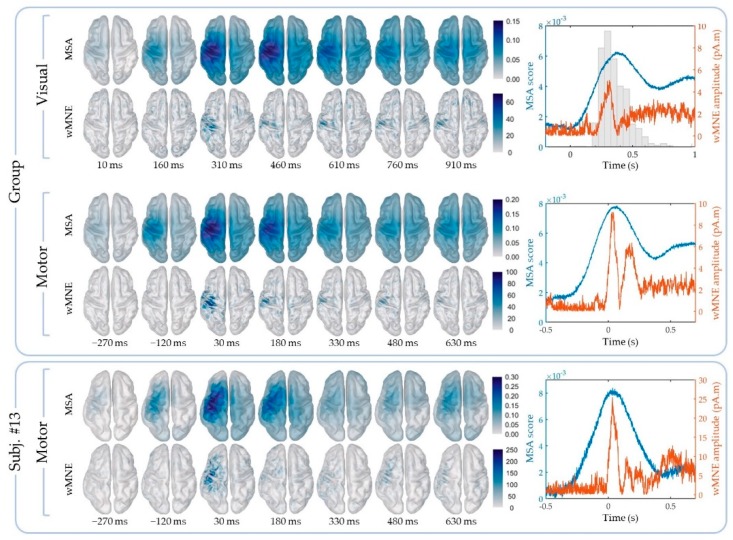
“FingerTap” experiment: (Left) Cortical mapping of the absolute values calculated using the multi-sensor space–time–frequency analysis (MSA) (upper rows) and the weighted minimum norm estimate (wMNE) method (lower rows). (Right) Time course of the mean absolute value in the Lmot region using the MSA (blue curve) and the wMNE method (orange curve). The additional histogram in the top-right plot indicates the participants’ response time after visual cue. The results shown correspond to the group-level calculations (upper frame) and to a specific subject for illustration (subj. #13, lower frame).

**Figure 4:**
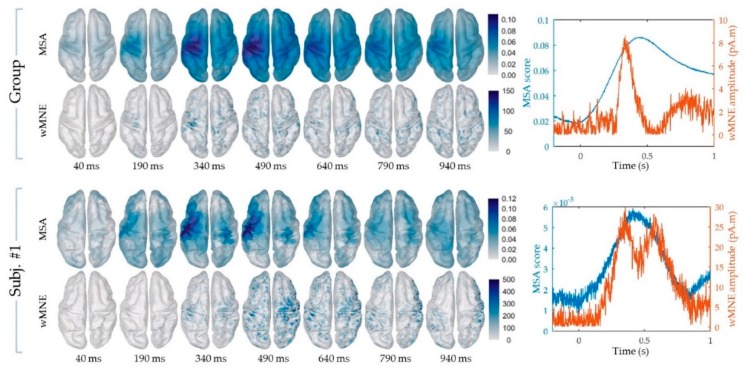
“FlexElbow” experiment: (Left) Cortical mapping of the absolute values calculated using the MSA (upper rows) and the wMNE method (lower rows), based on the visual trigger. (Right) Time course of the mean absolute value in the Lmot region using the MSA (blue curve) and the wMNE method (orange curve). The results shown correspond to the group-level calculations (upper frame) and to a specific subject for illustration (subj. #1).

The cortical maps of the absolute activity values were obtained for each participant and on average across participants using the MSA and wMNE methods for the “FingerTap” experiment (Figure 3, left) and the “FlexElbow” experiment (Figure 4, left). The mean absolute value over the vertices of the ten selected regions (Figure 2) was computed by participant and on average across participants. This allowed us to represent the time-course activity of a specific region such as the left motor region (“Lmot”) (Figure 3, right and Figure 4, right). Values exceeding the fifth quintile of the distribution value obtained from the ten regions of interest were considered as significant.

### 3.1. “FingerTap” Experiment

Brain activations during tapping were analyzed with respect to the motor trigger and the presentation of the visual cue. Because the task involved the right finger, brain activations were expected in the left motor region. 

#### 3.1.1. Tapping: Motor Triggering

**Figure 5:**
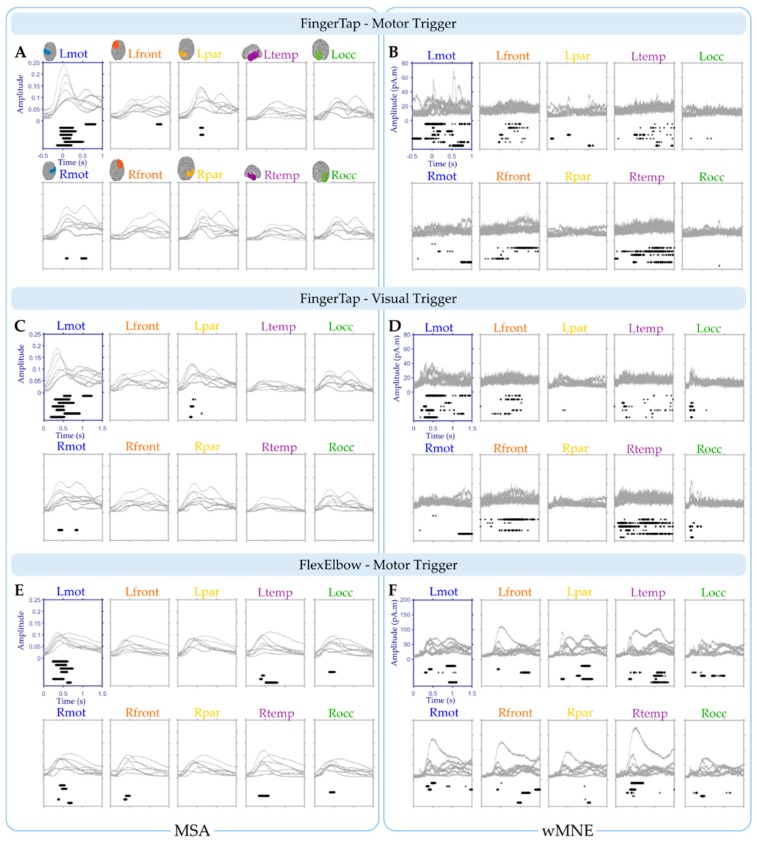
Participants’ mean absolute value of activity for the ten regions of interest using the MSA (MPC values) (**A**,**C**,**E**) and the wMNE method (current values) (**B**,**D**,**F**). Brain activations were triggered on the motor response (**A**,**B**) and on the visual cue (**C**,**D**) for the “FingerTap” experiment and on the visual cue only for the “FlexElbow” task (**E**,**F**). Each grey curve corresponds to the values obtained for a given participant during a specific task. For each region, significant time points are shown by black asterisks displayed below the curves (one row by participant).

Using the MSA method (Figure 5A), four regions: Lmot, Lfront, Lpar and Rmot exhibit at least one significant time point over all the participants. Only one participant was found with significant values for the Lfront and Rmot regions: participant 1 and 5 respectively. For the Lpar region, two participants: participants 2 and 4 were found with significant time points. For the Lmot region however, significant time points were found for all the participants. On average across participants, 84.5% (SD = 17.7, range: 55–100%) of the significant time points were found in the Lmot region with an average median timing of 0.199 s (SD = 0.23, range: 0.038–0.698 s). This shows the selectivity of the MSA method. 

The same analysis was performed using the wMNE method (Figure 5B). Significant time points were found for the all ten regions. In particular, for the Lmot region all the participants exhibit significant time points with an average median timing of 0.223 s (SD = 0.38, range: −0.368–0.717 s). Overall, 46.8% (SD = 30.5, range: 9–92%) of the significant time points were found in the Lmot region on average across participants. This value was significantly lower than the 84.5% obtained using the MSA method (Wilcoxon signed rank test, one-tail, *p* = 0.039).

The MSA results obtained on the motor trigger show that this method can reliably track the increase of activations in the left motor cortex elicited by the right finger tapping. Indeed, most of the significant time points were observed in the left motor cortex around the onset of the movement. This spatial and temporal selectivity was not as strong when using the wMNE analyses. In the following part, we test whether the MSA method is still robust when brain activations are triggered on the visual cue. 

#### 3.1.2. Tapping: Visual Triggering

Tracking brain activations on the visual cue onset is associated with some uncertainty regarding the starting time point of the executed movement. This trial-to-trial variability does not hold when the brain activations are epoched in relation to the motor onset. We tested whether the MSA remains a robust method in such conditions. The same methodology used with the motor trigger is applied 

Using the MSA method (Figure 5C), two regions: Lpar and Rmot exhibit at least one significant time point over all the participants. Only one participant was found with significant values for the Rmot regions: participant 5 and four participants for the Lpar region: participants 2, 4, 6 and 7. For the Lmot region however, significant time points were found for all the participants. On average across participants, 89.9% (SD = 16.6, range: 56–100%) of the significant time points were found in the Lmot region with an average median timing of 0.556 s (SD = 0.24, range: 0.348–1.026 s). 

The same analysis was performed for the data obtained with the wMNE method (Figure 5D). Significant time points were found for nine regions. In particular, for the Lmot region six participants exhibit significant time points with an average median timing of 0.641 s (SD = 0.35, range: 0.296–1.218 s). Overall, 34.4% (SD = 35.9, range: 0–92%) of the significant time points were found in the Lmot region on average across participants. This percentage was significantly lower than the 89.9% observed using the MSA method (Wilcoxon signed rank test, one-tail, *p* = 0.0078). 

Brain activations during right tapping movements could be tracked with high selectivity in the left motor region using the MSA method compared to the classical wMNE method. The difference between the two methods was particularly visible when the movements were triggered on the visual cue onset. According to our data, the MSA method offers a better robustness in such conditions. In the next experiment we will test whether the MSA method performs well to detect brain activations elicited by right elbow flexions epoched on a visual cue.

### 3.2. “FlexElbow” Experiment

Overall, seven regions of interest show significant time points for at least one of the participants using the MSA method (Figure 5E). For the wMNE method, significant values were found for all the ten regions (Figure 5F). In the Lmot region, significant time points were obtained for six participants with an average median timing of 0.498 s (SD = 0.10, range: 0.376–0.642 s) for the MSA method. For the wMNE method, only four participants exhibit significant time points in the Lmot region with an average median timing of 0.674 s (SD = 0.35, range: 0.318–1.006). The percentage of significant time points in the Lmot region was higher using the MSA method (54.4%, SD = 37.6, range: 0–100%) compared to the wMNE method (14.7%, SD = 19.0, range: 0–47.5%) (Wilcoxon signed rank test, one-tail, *p* = 0.031).

Again, the MSA method shows higher performance than the wMNE method to track selectively activations induced by specific movements. By replicating the results observed in the “FingerTap” experiment on the visual trigger, the MSA method provides a solid framework to identify critical brain activation features in the space–time domain.

The Table 1 summarizes the results obtained in the Lmot region.

The confidence in the obtained results can be evaluated using the margin of error (*MOE*) of our population (*n* = 14) which follows a binomial distribution as it involves a binary response: the Lmot region was found significant for 13 participants (*x* = 13). To estimate this *MOE* we used the Adjusted Wald method proposed by Agresti and Coull [36]:

$$MOE=Z\sqrt{V’}$$

where $V^{\prime }=p’\left(1-p^{\prime }\right)/\left(n+4\right)$ is the estimated population variance, $p^{\prime }=\left(x+2\right)/\left(n+4\right)$ is the sample proportion, and *Z* = 1.96 for a 95% confidence interval.

This provides a reliable estimate of the MOE when the sample size (*n*) is small and the sample proportion (*p’*) is close to extreme values (0 or 1). The resulting *MOE* was 0.17. Applying this *MOE* to our experimental observations: (1−MOE)·(x/n)=0.77. This means that we can be 95% sure that the MSA method works at least 77% of the time. This result provides a reliable estimate of the effectiveness of the MSA method.

### 3.3. Group-Level Analysis

**Figure 6:**
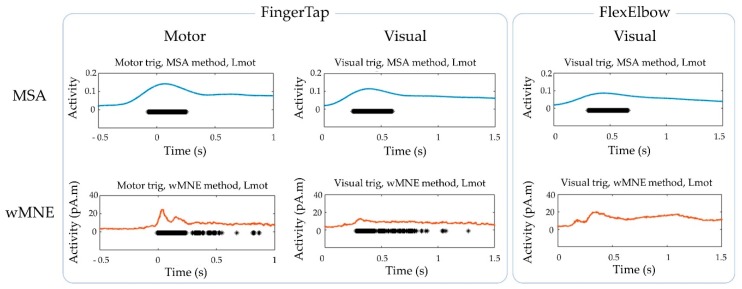
Group-level statistics for the “FingerTap” and the “FlexElbow” experiments. Significant time points obtained in the Lmot region on average across participants using the MSA and wMNE methods.

The data obtained on average across participant were used to compare the MSA and wMNE methods at the group level (Figure 6). For the “FingerTap” experiment the percentage of significant time points in the Lmot region was high for both the MSA: 88.5% and 92.3% and the wMNE methods 84.3% and 93.3% for the motor and visual triggers respectively. For the MSA method, the median timing of the significant time points in the Lmot region was 0.091 s and 0.423 s for the motor and visual triggers respectively. For the wMNE method, these values were 0.169 s and 0.445 s respectively. For the “FlexElbow” experiment, 94.4% of the significant time points (median timing = 0.479 s) were found in the Lmot region when using the MSA method whereas no significant time points were found in this region with the wMNE approach.

## 4. Discussion

This paper proposes a simple MSA statistical approach to overcome, to some extent, the shortcomings of MEG data analysis approaches which are currently widely used. These shortcomings include limited frequency resolution, difficulties in processing multi-sensor/multi-frequency data and sensitivity to ill-defined triggers. MSA uses cross-validated shifted multiple Pearson correlation, calculated from the TF transformed signal of brain activity (using co-localized sensors) and the binary signal of stimuli, for estimating task-specific neural activations. The MSA statistical approach was compared to the wMNE method widely used in MEG data analysis. The percentage of significant time points found in the Lmot region was used as an index of spatial selectivity. Our results from two motor experiments show that the MSA approach provides higher spatial selectivity compared to source imaging method. This difference was particularly important when considering visual triggers. Indeed, when using the visual cues, the exact timing of the motor response onset is prone to trial-to-trial variation. Such a latency-jitter phenomenon would affect methods that are based on trial averaging such as the wMNE. This means that the MSA approach mainly localized the task correlated activity over the motor cortical region in the time window used for this analysis, and revealed less activity outside the contralateral SM cortex. While the wMNE method localized source over the motor cortex, it also localized source over other cortical regions. Indeed, the wMNE method computes sources in order to explain, as completely as possible, the brain signal, solving ill-posed inverse problem. Thus, wMNE results are sensitive to artifacts or noise in the data, despite the averaging step, which seeks to increase the signal-to-noise ratio. The higher performance observed for the MSA approach could result from the more stable computing compared to wMNE. Indeed, as it does not rely on epoch averaging, MSA is less sensitive to the signal nulling induced by a jitter in the trigger, particularly at high frequencies. Hence, MSA allows the proper analyzes more efficiently high frequency content of input data. 

The goal of considering an MSA approach was to better take into account the frequency content of the brain signal, to integrate data from different kinds of sensors, and to improve robustness for a restricted dataset. Typically, we envision restrictions such as single-subject presurgical functional brain mapping for patients with motor disabilities where the subject condition avoids the use of motor trigger or lengthy acquisitions, making it impossible to follow clinical practical guidelines [37]. Source reconstruction methods such as wMNE are usually applied to averaged signals locked to the stimulus or the task of interest, as this is required to enhance the signal-to-noise ratio of data before modeling sources. Consequently, brain sources modeled with these source reconstruction algorithms are mainly based on low-frequency information contained in the recorded brain signal. The MSA approach has been designed to take into account broad frequency band information. Methodological efforts are currently undertaken to apply source reconstruction trial by trial, but this approach remains limited [38,39,40]. The MSA approach also enables one to combine signals from different kinds of magnetic sensors, such as gradiometers and magnetometers, which is more difficult with mathematical and biophysical approaches used in source reconstruction.

In terms of timing, the group-level analysis showed good accordance between the latencies measured with both methods: ~0.100 s for the motor trigger and ~0.450 s for the visual trigger. These timings are coherent with the cues that are considered. 

Several methodological comparisons between various source imaging approaches have already been reported [4,41,42]. In this study, the MSA approach is mathematically different from the wMNE method. This differentiates this work from previous methodological comparison studies. MSA is not an inverse problem-solving algorithm, such as source reconstruction methods, e.g., wMNE. Rather, it is a statistical approach of brain signal processing in the sensor space. Therefore, in terms of localization over the cortical surface, MSA requires one to project data over the cortical surface, for a given brain mesh point. In this way, the cortical images from MSA do not correspond to biophysical modeling of current densities (and inverse problem solving), as performed with source reconstruction. The counterpart is that anatomo-functional localization may be poorer than that obtained from source reconstruction using 15,000 dipoles covering the brain surface (as with wMNE). This issue requires further study. In addition, applying MSA to current densities over the brain surface, computed from each trial and each gradiometer or magnetometer, should work around this issue; however, it would require high computational resources. The advantage is that MSA provides unicity of the mathematical estimate of task-correlated cortical activity, and is intrinsically more stable and more robust for a restricted dataset. Thus, possible poorer localization (in term of spatial accuracy) but better spatial selectivity and more stability, would be preferred in some cases; such cases would favor the MSA approach rather than source reconstruction. 

An important advantage of the MSA method is its robustness. The numerous preprocessing steps needed for reliable source reconstruction improving the signal-to-noise ratio such as trial inspection/rejection, and ICA decomposition followed by manual component suppression, are not necessary and were not applied when performing MSA calculations in the present study. The MSA method is applied in a completely automatic manner and thus is less sensitive to user qualification/experience.

The motor tasks with a visual cue as the trigger were chosen in the current study as prospective tasks to be used in brain computer interface (BCI) applications of four-limb exoskeleton control [8], in patients with tetraplegia. The “FlexElbow” motor task is typical for BCI paradigms and our results show that our method can be used efficiently on single subjects to localize activity related to this task. 

An important perspective is the exploration and testing of both MSA and wMNE approaches in subjects with motor disabilities (e.g., persons with paraplegia and tetraplegia). In these patients, movement attempt-related brain activity should be analyzed. The methods are currently being applied to MEG data from both healthy subjects and patients, in a clinical trial (reference number NCT02574026) which focuses on functional mapping in various real and imaginary movements. Further study will bring new insights into the respective advantages of the MSA approach compared to source reconstruction. 

## 5. Conclusions

All in all, the MSA is a method for multimodal analysis of multi trial recordings from co-localized (possibly different) sensors and with informative content in temporal and frequency domains. The example of application is sensorimotor cortex localization using MEG recording with a pair of gradiometers. A regression-based MSA integrates multi-sensors and multi-frequency information to estimate task-specific brain activations using cross validated, shifted, multiple Pearson correlation. The proposed method was compared to the benchmark wMNE source imaging method using MEG recordings performed in fourteen subjects during two motor tasks. The MSA approach provided good localization performance when compared to wMNE and a statistically significant improvement of robustness against ill-defined triggers. The improvement of robustness may be especially important in the use case of patients with motor deficits, when synchronization of trials is uncertain. In conclusion, the MSA statistical approach can be preferable to analyze a restricted MEG dataset if the frequency content of the signal is essential, and stability of the localization solution is sought. Further study is needed to clarify noise steadiness and characteristics depending on the amount of data.

The proposed method may be useful for other applications of multimodal analysis of multi trial experiments with moderate uncertainty in trials synchronization, e.g., in the fields of telecommunications, earthquake detection, or medical ultrasound imaging (wavefront correction).

## Figures and Tables

**Table 1:** Results obtained for the Lmot region for the MSA and wMNE methods on three experimental conditions.

	MSA			wMNE		
Experimental Condition	Participants	Time Points (%)	Timing (s)	Participants	Time Points (%)	Timing (s)
Motor trigger						
FingerTap	7/7	84.5 *	0.199	7/7	46.8	0.223
Visual trigger						
FingerTap	7/7	89.9 **	0.556	6/7	34.4	0.641
FlexElbow	6/7	54.4 *	0.498	4/7	14.7	0.674

* *p* < 0.05, ** *p* < 0.01.

## References

[1] Baillet S. (2017). Magnetoencephalography for brain electrophysiology and imaging. Nat. Neurosci..

[2] Tikhonov A.N., Arsenin V.I. (1977). Solutions of Ill-Posed Problems.

[3] Baillet, Mosher, Leahy Electromagnetic Brain Mapping. http://cogimage.dsi.cnrs.fr/hmtc/references/files/BailletMosherLeahy_IEEESPMAG_No.pdf.

[4] Becker H., Albera L., Comon P., Haardt M., Birot G., Wendling F., Gavaret M., Bénar C.G., Merlet I. (2014). EEG extended source localization: Tensor-based vs. conventional methods. Neuroimage.

[5] Gramfort A., Kowalski M., Hämäläinen M. (2012). Mixed-norm estimates for the M/EEG inverse problem using accelerated gradient methods. Phys. Med. Biol..

[6] Wipf D., Nagarajan S. (2009). A unified Bayesian framework for MEG/EEG source imaging. Neuroimage.

[7] Hansen P.C., Kringelbach M.L., Salmelin R. (2010). MEG: An Introduction to Methods.

[8] Benabid A.L., Costecalde T., Eliseyev A., Charvet G., Verney A., Karakas S., Foerster M., Lambert A., Morinière B., Abroug N. (2019). An exoskeleton controlled by an epidural wireless brain–machine interface in a tetraplegic patient: A proof-of-concept demonstration. Lancet Neurol..

[9] Miao A., Xiang J., Tang L., Ge H., Liu H., Wu T., Chen Q., Hu Z., Lu X., Wang X. (2014). Using ictal high-frequency oscillations (80-500Hz) to localize seizure onset zones in childhood absence epilepsy: A MEG study. Neurosci. Lett..

[10] Grent T., Rivolta D., Sauer A., Grube M., Singer W., Wibral M., Uhlhaas P.J. (2016). MEG-measured visually induced gamma-band oscillations in chronic schizophrenia: Evidence for impaired generation of rhythmic activity in ventral stream regions. Schizophr. Res..

[11] von Ellenrieder N., Pellegrino G., Hedrich T., Gotman J., Lina J.-M., Grova C., Kobayashi E. (2016). Detection and Magnetic Source Imaging of Fast Oscillations (40–160 Hz) Recorded with Magnetoencephalography in Focal Epilepsy Patients. Brain Topogr..

[12] Xiang J., Luo Q., Kotecha R., Korman A., Zhang F., Luo H., Fujiwara H., Hemasilpin N., Rose D.F. (2014). Accumulated source imaging of brain activity with both low and high-frequency neuromagnetic signals. Front. Neuroinform..

[13] Gummadavelli A., Wang Y., Guo X., Pardos M., Chu H., Liu Y., Horn P., Zhang F., Xiang J. (2013). Spatiotemporal and frequency signatures of word recognition in the developing brain: A magnetoencephalographic study. Brain Res..

[14] Ramirez R.R., Kopell B.H., Butson C.R., Gaggl W., Friedland D.R., Baillet S. (2009). Neuromagnetic Source Imaging of Abnormal Spontaneous Activity in Tinnitus Patient Modulated by Electrical Cortical Stimulation. Proceedings of the 2009 Annual International Conference of the IEEE Engineering in Medicine and Biology Society.

[15] Tan H.-R.M., Gross J., Uhlhaas P.J. (2015). MEG—Measured auditory steady-state oscillations show high test–retest reliability: A sensor and source-space analysis. NeuroImage.

[16] De Lange F., Jensen O., Bauer M., Toni I. (2008). Interactions between posterior gamma and frontal alpha/beta oscillations during imagined actions. Front. Hum. Neurosci..

[17] Düzel E., Habib R., Schott B., Schoenfeld A., Lobaugh N., McIntosh A.R., Scholz M., Heinze H.J. (2003). A multivariate, spatiotemporal analysis of electromagnetic time-frequency data of recognition memory. Neuroimage.

[18] Kauhanen L., Nykopp T., Sams M. (2006). Classification of single MEG trials related to left and right index finger movements. Clin. Neurophysiol..

[19] Tadel F., Baillet S., Mosher J.C., Pantazis D., Leahy R.M. (2011). Brainstorm: A user-friendly application for MEG/EEG analysis. Comput. Intell. Neurosci..

[20] Yeom H.G., Kim J.S., Chung C.K. (2013). Estimation of the velocity and trajectory of three-dimensional reaching movements from non-invasive magnetoencephalography signals. J. Neural Eng..

[21] Gross J., Baillet S., Barnes G.R., Henson R.N., Hillebrand A., Jensen O., Jerbi K., Litvak V., Maess B., Oostenveld R. (2013). Good practice for conducting and reporting MEG research. NeuroImage.

[22] Taulu S., Simola J. (2006). Spatiotemporal signal space separation method for rejecting nearby interference in MEG measurements. Phys. Med. Biol..

[23] Fischl B. (2012). FreeSurfer. NeuroImage.

[24] Aguera P.-E., Jerbi K., Caclin A., Bertrand O. (2011). ELAN: A Software Package for Analysis and Visualization of MEG, EEG, and LFP Signals. Comput. Intell. Neurosci..

[25] Shimoda K., Nagasaka Y., Chao Z.C., Fujii N. (2012). Decoding continuous three-dimensional hand trajectories from epidural electrocorticographic signals in Japanese macaques. J. Neural Eng..

[26] Zhao Q., Zhang L., Cichocki A. (2009). EEG-based asynchronous BCI control of a car in 3D virtual reality environments. Chin. Sci. Bull..

[27] Eliseyev A., Aksenova T. (2016). Penalized Multi-Way Partial Least Squares for Smooth Trajectory Decoding from Electrocorticographic (ECoG) Recording. PLoS ONE.

[28] Draper N.R., Smith H. (1998). Applied Regression Analysis.

[29] Marquardt D.W., Snee R.D. (1975). Ridge Regression in Practice. Am. Stat..

[30] Shepard D. (1968). A Two-Dimensional Interpolation Function for Irregularly-Spaced Data. Proceedings of the 1968 23rd ACM National Conference.

[31] Gramfort A., Luessi M., Larson E., Engemann D.A., Strohmeier D., Brodbeck C., Parkkonen L., Hämäläinen M.S. (2014). MNE software for processing MEG and EEG data. NeuroImage.

[32] Oostenveld R., Fries P., Maris E., Schoffelen J.-M. (2011). FieldTrip: Open Source Software for Advanced Analysis of MEG, EEG, and Invasive Electrophysiological Data. Comput. Intell. Neurosci..

[33] Leahy R.M., Mosher J.C., Spencer M.E., Huang M.X., Lewine J.D. (1998). A study of dipole localization accuracy for MEG and EEG using a human skull phantom. Electroencephalogr. Clin. Neurophysiol..

[34] Huang M.X., Mosher J.C., Leahy R.M. (1999). A sensor-weighted overlapping-sphere head model and exhaustive head model comparison for MEG. Phys. Med. Biol..

[35] Fonov V., Evans A., McKinstry R., Almli C., Collins D. (2009). Unbiased nonlinear average age-appropriate brain templates from birth to adulthood. NeuroImage.

[36] Agresti A., Coull B.A. (1998). Approximate Is Better than “Exact” for Interval Estimation of Binomial Proportions. Am. Stat..

[37] Burgess R.C., Funke M.E., Bowyer S.M., Lewine J.D., Kirsch H.E., Bagić A.I. (2011). American Clinical Magnetoencephalography Society Clinical Practice Guideline 2: Presurgical Functional Brain Mapping Using Magnetic Evoked Fields*. J. Clin. Neurophysiol..

[38] Dubarry A.-S., Badier J.-M., Trébuchon-Da Fonseca A., Gavaret M., Carron R., Bartolomei F., Liégeois-Chauvel C., Régis J., Chauvel P., Alario F.-X. (2014). Simultaneous recording of MEG, EEG and intracerebral EEG during visual stimulation: From feasibility to single-trial analysis. NeuroImage.

[39] Lee P. (2003). ICA-based spatiotemporal approach for single-trial analysis of postmovement MEG beta synchronization⋆. NeuroImage.

[40] Lee P.-L., Shang L.-Z., Wu Y.-T., Shu C.-H., Hsieh J.-C., Lin Y.-Y., Wu C.-H., Liu Y.-L., Yang C.-Y., Sun C.-W. (2009). Single-Trial Analysis of Cortical Oscillatory Activities During Voluntary Movements Using Empirical Mode Decomposition (EMD)-Based Spatiotemporal Approach. Ann. Biomed. Eng..

[41] Grech R., Cassar T., Muscat J., Camilleri K.P., Fabri S.G., Zervakis M., Xanthopoulos P., Sakkalis V., Vanrumste B. (2008). Review on solving the inverse problem in EEG source analysis. J. Neuroeng. Rehabil..

[42] Soufflet L., Boeijinga P.H. (2005). Linear Inverse Solutions: Simulations from a Realistic Head Model in MEG. Brain Topogr..

